# High-precision information retrieval for rapid clinical guideline updates

**DOI:** 10.1038/s41746-025-01648-5

**Published:** 2025-04-27

**Authors:** Florian Borchert, Paul Wullenweber, Annika Oeser, Nina Kreuzberger, Torsten Karge, Thomas Langer, Nicole Skoetz, Lothar H. Wieler, Matthieu-P. Schapranow, Bert Arnrich

**Affiliations:** 1 Hasso Plattner Institute for Digital Engineering, University of Potsdam, Potsdam, Germany; 2 Institute of Public Health, Medical Faculty and University Hospital Cologne, University of Cologne, Cologne, Germany; 3 Clinical Guideline Services, Kiel, Germany; 4 Department of Gastroenterology, Infectious Diseases and Rheumatology, Charité - Universitätsmedizin Berlin, Berlin, Germany; 5 German Guideline Program in Oncology, German Cancer Society, Berlin, Germany; 6 Hasso Plattner Institute for Digital Health at Mount Sinai, Icahn School of Medicine at Mount Sinai, New York, NY USA

**Keywords:** Computer science, Information technology, Scientific data

## Abstract

Delays in translating new medical evidence into clinical practice hinder patient access to the best available treatments. Our data reveals an average delay of nine years from the initiation of human research to its adoption in clinical guidelines, with 1.7–3.0 years lost between trial publication and guideline updates. A substantial part of these delays stems from slow, manual processes in updating clinical guidelines, which rely on time-intensive evidence synthesis workflows. The Next Generation Evidence (NGE) system addresses this challenge by harnessing state-of-the-art biomedical Natural Language Processing (NLP) methods. This novel system integrates diverse evidence sources, such as clinical trial reports and digital guidelines, enabling automated, data-driven analyses of the time it takes for research findings to inform clinical practice. Moreover, the NGE system provides precision-focused literature search filters tailored specifically for guideline maintenance. In benchmarking against two German oncology guidelines, these filters demonstrate exceptional precision in identifying pivotal publications for guideline updates.

## Introduction

The past years have witnessed remarkable advances in biomedical Natural Language Processing (NLP), significantly enhancing the ability to extract meaningful insights from unstructured sources of medical evidence, including clinical trial reports and clinical guidelines^1,2^. While the NLP community has extensively studied primary research publications in the past, the potentials of applying NLP to international clinical guidelines remain under-explored. Recent innovations in multilingual and domain-specific medical language models have greatly improved the viability of using data from world-wide clinical guidelines in software systems and support the timely translation of clinical research into actionable recommendations for healthcare decision-making^3,4^.

Today, the translation of new evidence into clinical practice is hindered by multiple delays, as highlighted in various case studies across medical fields that manually reviewed publications^5–7^. As a prominent example, Hanney et al.^8^ performed an analysis of time lags across 11 calibration points in clinical research, finding widely varying time lags from *discovery* (basic research) to *implementation*, ranging from 18 years (early interventions for schizophrenia) to 54 years (smoking reduction).

A substantial factor contributing to these delays is the inherent complexity of clinical trials, which require extensive time for ensuring safety, efficacy, and robust data collection^9^. Yet, the volume of published research results in the primary medical literature is still so large, that another bottleneck becomes *evidence synthesis*, i.e., a summary of the body of evidence with a critical appraisal of its quality and impact for clinical practice^10^. A search in PubMed (using the query “Clinical Trial”[Publication Type]) reveals that approximately one million articles dealing with clinical trials are indexed in Medline as of March 2025, more than 30 thousand of them have been added in 2024 alone (about 82 per day). In effect, incorporating all available evidence into evidence synthesis workflows for guideline development becomes increasingly time-consuming.

In this work, we focus on the particular delay induced by current update protocols for clinical guidelines, i.e., the time it takes to incorporate successfully published research results into guideline recommendations. These protocols vary across guideline groups, but they usually involve a formulation of key questions using the Population–Intervention–Comparison–Outcome (PICO) framework, a systematic literature search, data extraction, assessment of the robustness of the underlying evidence, as well as procedures to arrive at recommendations through evidence-to-decision (EtD) frameworks like GRADE and structured consensus-finding processes^11–13^. For literature retrieval in these projects, most guideline developers follow similar approaches, like Boolean searches in literature databases such as PubMed^14^. These searches tend to aim for near-perfect *recall*, while suffering from notoriously low levels of *precision*, i.e., most search results are irrelevant^15^. Common search queries may return thousands of results, which need to be reviewed manually by human experts through screening of title, abstract, and full-text^16^. Although there is an growing body of research attempting to automate parts of the process^17,18^, many of these research prototypes are not yet widely adopted in practice due to their lack of validation. Recently, *living guidelines* have gained attention, aiming for continuous updates at the level of individual recommendations as new evidence emerges^19^. A natural implementation of such a surveillance strategy would be the regular application of an existing search query to the stream of newly published literature^20^. However, this would not alter the overall screening burden incurred by low retrieval precision of existing search strategies.

Assuming that comprehensive (manual or semi-automated) literature reviews within the scope of a guideline topic are performed at regular intervals, all relevant publications should be covered by such a review at some point in time. Thus, a complementary search strategy aiming for high precision instead of perfect recall can focus on *signal publications*, i.e., publications indicating new evidence likely to significantly impact guideline recommendations, thus warranting a timely investigation by guideline developers. Shekelle et al.^21^ describe such an approach, based on “limited literature searches and expert opinion” in the context of systematic reviews. The American Society of Clinical Oncology (ASCO), has adopted a similar strategy for updating oncology guidelines, relying on “targeted literature searching and the expertise of ASCO guideline panel members”^22^. For example, ASCO has recently issued a rapid update of the non-small-cell lung cancer guideline based on the results of a single phase III randomized controlled trial (RCT) trial^23,24^.

This work presents a data-driven approach to address the challenges of intermittent clinical guideline updates, supported by an automated, NLP-enabled integration of diverse sources of primary and synthesized evidence. We propose the *Next Generation Evidence* (NGE) system, that relies on various innovative NLP components for structured information extraction from clinical trial reports, and clinical guidelines, developed in the context of the GGPOnc and xMEN projects^25–27^. Our harmonized database ensures semantic interoperability by mapping all relevant information from primary and synthesized evidence in different languages to Concept Unique Identifiers (CUIs) from the Unified Medical Language System (UMLS)^28^.

**Fig. 1:**
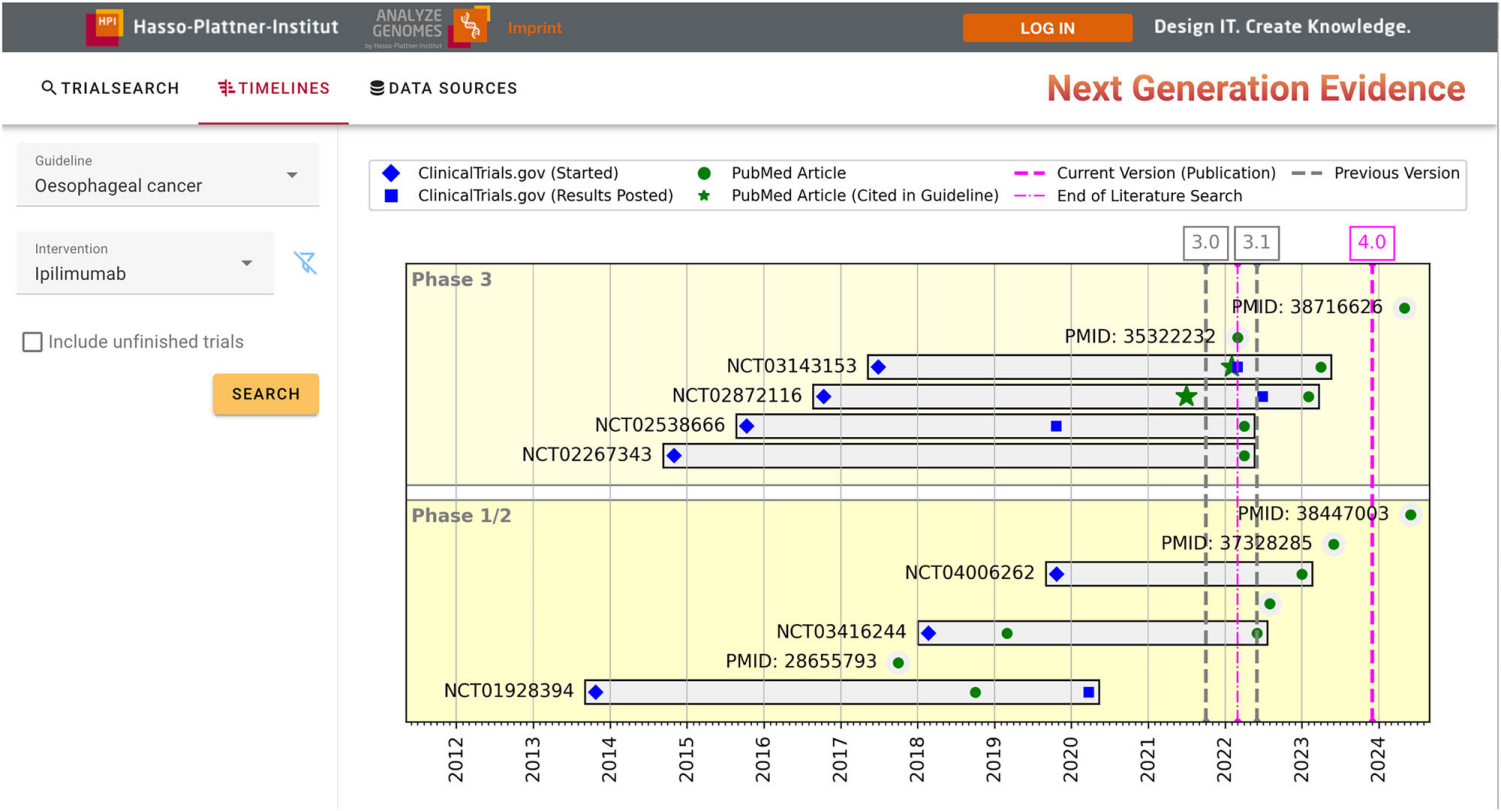
Timeline view of the NGE browser. The timeline view groups registered clinical trials from ClinicalTrials.gov in terms of their start date, result publication data, and potential PubMed articles referencing this data on a horizontal timeline. Update intervals for the corresponding guideline are included as vertical lines.

**Fig. 2:**
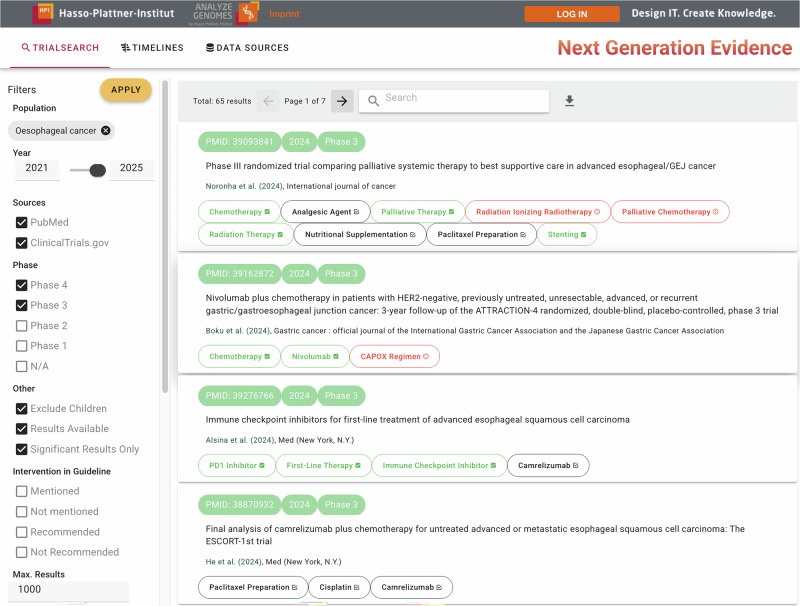
User interface for searching the NGE database for trials by population in combination with various filtering criteria. For all results, the system lists the identifier (PubMed or ClinicalTrials.gov ID), publication date, phase (I–IV), and title. For PubMed results, bibliographic information (authors and journals) is included. Extracted interventions are color-coded, and distinguishable with icons and tooltips, based on their occurrence in the guideline corresponding to the selected publication: black ones (outlined checkbox icon) are already mentioned (anywhere) in the guideline, green ones (filled checkbox) are mentioned within recommendations, red ones (exclamation mark) are mentioned nowhere. The results can also be downloaded as an Excel file for further analysis.

Moreover, we provide a user-friendly web application to interact with the database, shown in Figs. 1 and 2, which is also available online: https://we.analyzegenomes.com/nge/. We believe that our system can be useful for numerous stakeholders. Amongst others, guideline developers can use it (a) to implement targeted signal search strategies for clinical trial publications, e.g., for entirely new treatment options, (b) to quality-check the results of traditional searches, but also (c) to support existing (consensus-based) recommendations with more solid evidence. Furthermore, our tool can be used by guideline users to identify newly published evidence that might affect the interpretation of current recommendations prior to a guideline update^29^. While many existing systems, including the Trip database^30^ and various research prototypes^31–33^, offer advanced search options, the NGE system is, to the best of our knowledge, the first to enable contextualized searches with respect to current clinical practice by prioritizing interventions not yet included in guidelines.

We evaluate our system experimentally as follows. First, we perform an analysis across the integrated data sources with the goal to automatically estimate the time it takes to translate research on new treatments for cancer patients from the first clinical trials with human subjects to recommendations in oncology guidelines. Second, we show how the system can be used to identify signal publications, which might be relevant for prospective guideline updates. To avoid an unreasonable increase in the screening workload induced by existing search strategies (which our system complements rather than replaces), our approach aims for maximal precision, i.e., any retrieved publication should be relevant for guideline developers and users with high probability. Two recent updates to German oncology guidelines are used for evaluating retrieval performance in a real-world setting: (1) *oesophageal cancer* and (2) *Hodgkin lymphoma*. The data from these updates enables an assessment of precision and recall in comparison with established guideline update protocols.

## Results

In this section, we give an overview of the data integrated within the NGE database. Furthermore, we show how this data is used to a) estimate time lags in research translation and to b) retrieve signal publications for guideline updates.

### The NGE database

Table 1 shows the total number of documents for each data source in the NGE database, as well as the total and unique numbers of population and intervention concepts. The seed UMLS CUIs for the 34 guideline topics are expanded to more than 17K population CUIs (please refer to the Methods section for details on the concept mapping). Although most concepts are unique, there is a certain degree of overlap in the subtrees descending from the root population concepts (the UMLS hierarchy allows a concept to have multiple direct ancestors). As an example, cancers of the *oesophagogastric junction* are partially covered by both the guideline for *gastric cancer* and the guideline for *oesophageal cancer*. The statistics also indicate that RCT reports and registered trials cover a much higher number of populations and interventions than guidelines and CIViC because the integrated clinical trial data is not limited to cancer patients. The relative number of unique CUIs in PubMed is much lower: the most frequently occurring CUIs belong to very general concepts, e.g., “patient”, “women”, “adult”, “neoplasm” (populations), or “treatment”, “placebo”, “administration”, “antineoplastic agent” (interventions). The numbers of unique concepts in ClinicalTrials.gov and CIViC are particularly low; presumably, because codes have been assigned by human curators based on controlled vocabularies rather than being automatically extracted from natural language text.

**Table 1:** Overview of harmonized information for all integrated data sources in the NGE database

Data Source	Guidelines (GGPO CMS)	PubMed (RCT Reports)	ClinicalTrials.Gov (Registered Trials)	CIViC (POKB)
Date yyyy-mm-dd	2024-06-18	2024-07-17	2024-06-30	2024-07-17
Documents	34	828,356	499,882	10,745
Populations	Topics	Population	Condition	Pheno./ Dis.
Total	17,530	7,326,622	3,404,118	15,919
Unique CUIs	12,005	52,568	4466	545
Interventions	Drugs/ Proc.	Interventions	Interventions	Therapies
Total	129,119	5,631,219	1,543,754	6273
Unique CUIs	15,852	68,738	3994	418

Currently, the database includes (1) digital clinical guidelines extracted from the content management system (CMS) of the German Guideline Program in Oncology (GGPO), (2) abstracts and metadata of randomized controlled trials (RCTs) from PubMed, (3) registered clinical trials from ClinicalTrials.gov, as well as (4) curated assertions from CIViC, as a precision oncology knowledge base (POKB) for the clinical classification of cancer variants. As the data in guidelines and RCT reports is reported as natural language text, we apply various NLP components for extracting structured information (described in the Methods section). All results refer to data imported on July 17th, 2024, which was used for our evaluation. Data in the production system differs as it is regularly updated.

### Time lags in research translation

To evaluate time lags in translation from primary research to clinical guideline recommendations, we consider as input data all updates to the German Guideline Program in Oncology (GGPO) guidelines for the years 2022–2024. 12 guidelines (out of 34 maintained by the GGPO) received an update during the considered time frame, and a few have been updated multiple times (15 updates in total, including minor updates).

**Fig. 3:**
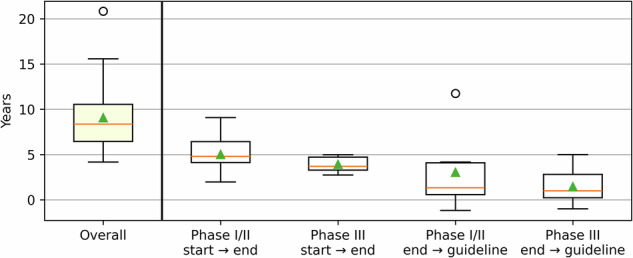
Box plot showing the distribution of time lags between different points in the evidence translation process across all newly recommended interventions (*n* = 22). The horizontal line within each box represents the median, the upper triangle the mean. The *Overall* time span distribution (leftmost plot) covers the time from the start date of the first trial in humans until recommendation in the respective guideline, with a mean value of 9.05 years, and a maximum of 20.8 years. The remaining boxplots show subsets of this timeline.

In total, 22 new interventions across oncology guideline updates could be identified. All of these were manually validated as referring to new interventions in the focus of the respective guideline update. A detailed list of interventions and corresponding guideline updates is provided in Supplementary Table 1. Figure 3 shows a box plot of the distribution of time lags for all these interventions. The average time from the start of the first human trial to inclusion in a guideline recommendation is approximately nine years, which aligns with an estimate of eight to ten years by Subbiah^9^. As some guideline updates are based on results of phase I/II clinical trials, interventions with and without phase III trials are shown separately. When a guideline recommendation is based on the results of a phase I or II clinical trial, it takes an average of three years from publication to guideline recommendation. In contrast, this time span is reduced to 1.7 years for recommendations based on results of phase III clinical trials; presumably, because results from a large phase III trial might be a strong motivation for updating a guideline in the first place.

The standard deviation on all reported values is relatively large. The most visible outlier refers to one case, when a clinical guideline included results of a trial almost *12 years* after its publication. This contrasts with a few cases where a recommendation has been made even *before* the results of an ongoing clinical trial were published (negative values on the y-axis). Moreover, the visualized time frames refer to *publication* of results. Hence, phase III trials are frequently started before phase I/II results are available in the literature, potentially owing to the (well-known) delays in academic publishing. A qualitative description of example data points from Fig. 3 is provided in Supplementary Figs. 1–4.

### Retrieval of signal publications

To reduce the delay between publication of clinical trial results and inclusion in guidelines, the NGE system can be used to implement a targeted literature search approach. Adding increasingly strict filter combinations allows users to balance between desired levels of precision and recall (please refer to the Methods section for details on the available filters). The utility of the NGE system for targeted literature searches is evaluated using two datasets of published evidence, screened by human experts for recent guideline updates: (a) the recently completed update of the German *oesophageal cancer* guideline (from version 3.1 to version 4.0) and (b) the ongoing update of the *Hodgkin lymphoma* guideline (from version 3.2 to version 4.0). These datasets cover the inclusion and exclusion decisions during literature screening and were generously provided by the respective guideline working groups as an export from their currently used literature management tools. Details on these datasets are shown in Table 2, with an in-depth description provided in the Methods section. All results presented in the following refer to the retrieval of individual PubMed articles, as our evaluation datasets contain only article-level screening decisions.

**Table 2:** Constructing two evaluation datasets for guideline updates through different steps of the literature screening process (starting after title–abstract screening for Hodgkin lymphoma)

	(a) Oesophageal Cancer	(b) Hodgkin Lymphoma
Minimum Publication Date	09/01/2019	01/01/2016
Search Date	03/04/2022	06/01/2023
Screened	3147	–
− Duplicates	139	–
= Unique references	3008	–
− *title–abstract excluded*	2741	–
= title–abstract included	267	168
− *Full-text excluded*	195	105
+ *Excluded, but already in guideline*	9	2
= Included (Evaluation)	81	65
⌞ RCTs included	26	25
+ Manual review	9	6
+ Retrieved, already in guideline	–	9
= **RCTs included (final)**	**35**	**40**
⌞ Other included	55	40
⌞ *Excluded (Evaluation)*	2927	103
⌞ *RCTs excluded*	290	24
+ *Manual review*	6	11
= ***RCTs excluded (final)***	**296**	**35**
⌞ Other excluded	2637	79

The lines marked in **bold** are used as the ground-truth for evaluating RCT retrieval for guideline updates. For instance, the *recall* of a search query is maximized when it can retrieve all of the 35 RCT publications that are considered for the oesophagael cancer guideline update.

There are instances of results retrieved by the NGE system, which were not included in the initial screening at all. Such references were sent to the two groups that conducted the literature screening, asking them for additional feedback on the relevance of these results. However, only RCTs after phase II were subject to manual review to limit the workload because these were supposedly more likely to be relevant. For these references, human subject-matter experts deemed nine out of 15 results (60.0%) potentially relevant for oesophageal cancer and six out of 17 results (35.3%) for Hodgkin lymphoma. The assigned inclusion and exclusion reasons for these references are shown in Table 3.

**Table 3:** Inclusion and exclusion reasons for manually reviewed references

Manual Review	(a) Oesophageal Cancer	(15)	(b) Hodgkin Lymphoma	(17)
Included	Potentially relevant	(3)	Potentially relevant	(6)
	Drug not in PICO search	(6)		
Excluded	Wrong population	(1)	Wrong population	(5)
	Wrong publication type	(1)	Wrong publication type	(6)
	Not in scope of update	(4)		

These correspond to the “Manual review” lines in Table 2.

For Hodgkin lymphoma, four out of six relevant references (66.7%) were actually part of the (temporally overlapping) literature search for the previous guideline version, but ultimately not considered by the guideline expert panel. Two references were erroneously excluded during title–abstract screening, the data of which is missing from the evaluation dataset for Hodgkin lymphoma. Regarding the oesophageal cancer guideline, where complete data is available, six references were not found originally, as the investigated drug was not explicitly part of the PICO (Boolean) search string. Regarding irrelevant results, the most common reason was a mismatch between the population and the scope of the guideline or the particular questions for the update. For instance, three results for Hodgkin lymphoma concerned children, which are out of scope of the guideline. Moreover, some results were indexed as RCTs in PubMed, but were, in fact, other publication types, e.g., secondary analysis of RCT data. The references that underwent a manual review were added to the final evaluation dataset as shown in Table 2.

Using this final evaluation dataset with comprehensive information on the relevance of retrieved trials, the impact of adding increasingly strict filters supported by the NGE data can be assessed. The results are shown in Table 4. First, all retrieved results after filtering for clinical phases after phase II can be classified as either true positives (TP) or false positives (FP), i.e., there are no “?” entries after the second row for each data subset. Second, high levels of precision can be achieved by adding increasingly strict filtering criteria. Most gains in precision over the baseline (just filtering by populations) can already be achieved by selecting RCTs with phase III or later, resulting in +18pp. for oesophageal cancer and +13pp. for Hodgkin lymphoma. Excluding children does not affect the result set for oesophageal cancer, but improves precision by another 6pp. for Hodgkin lymphoma.

**Table 4:** Combination of different filters and impact on precision for the Hodgkin lymphoma and oesophageal cancer guideline update

Search Queries	Retr.				¬ Retr.		Metrics		
(a) Oesophageal Cancer	Total	TP	?	FP	TN	FN	Pr.	Re.	F_1_
All RCTs	209	31	32	146	150	4	0.18	**0.89**	0.29
⌞ Phase ≥ II	94	27	**0**	67	229	8	0.29	0.77	0.42
⌞ Phase ≥ III	55	20	0	35	261	15	0.36	0.57	**0.44**
⌞ Excl. children	55	20	0	35	261	15	0.36	0.57	**0.44**
⌞ Significant result	37	15	0	22	274	20	0.41	0.43	0.42
⌞ ∃ Known interv.	31	14	0	17	279	21	**0.45**	0.40	0.42
⌞ ∃ Unknown interv.	28	12	0	16	280	23	0.43	0.34	0.38
**(b) Hodgkin Lymphoma**									
All RCTs	80	40	25	15	20	0	0.73	**1.00**	**0.84**
⌞ Phase ≥ II	45	32	**0**	13	22	8	0.71	0.80	0.75
⌞ Phase ≥ III	28	24	0	4	31	16	0.86	0.60	0.71
⌞ Excl. children	25	23	0	2	33	17	0.92	0.57	0.71
⌞ Significant result	10	10	0	0	35	30	**1.00**	0.25	0.40
⌞ ∃ Known interv.	10	10	0	0	35	30	**1.00**	0.25	0.40
⌞ ∃ Unknown interv.	10	10	0	0	35	30	**1.00**	0.25	0.40

There are no remaining results with unknown relevance (column “?”) for clinical trials after phase II.

Best values per column are highlighted in bold.

Adding a filter to retain only significant results achieves perfect precision on the Hodgkin lymphoma dataset: all results retrieved by the system are relevant for the guideline update, although with an overall recall of only 25.0%. For oesophageal cancer, additional gains can be obtained by filtering for trials with at least one known intervention, i.e., one that is mentioned in the guideline, or at least one unknown intervention. Interestingly, the best precision for oesophageal cancer is achieved when considering only clinical trials, which have at least one *known* intervention.

## Discussion

A key strength of the system is its demonstration of the utility of machine-readable and semantically interoperable guidelines, showcased through two use cases: time lag analysis and evidence retrieval for guideline updates.

The analysis of time lags has shown how important the right time point for updating a guideline can be. If not considered carefully, key results may be missed out in an update cycle. Building on that finding, we investigated how the NGE system can be used to retrieve potentially practice-changing signal publications at any given point during a guideline’s lifetime.

Our evaluation suggests that targeted literature searches can be implemented through increasingly precision-oriented filtering criteria. Combining structured metadata, extracted population concepts, and information about the trial phase already provides comparatively high levels of precision. In both evaluation scenarios, precision could be further increased by classifying publications according to the statistical significance of their results. Including guideline context, i.e., which interventions are already recommended, can increase precision even further.

The relatively high number of results that were neither marked as included nor excluded in the provided screening dataset is rather surprising. This finding suggests the utility of our NGE system for quality-control in the systematic review processes, which has the goal of maximizing recall while ensuring reproducibility. This investigation should be repeated prospectively with a larger set of guidelines. For instance, if the system finds results not included in the original screening set due to the nature of the employed Boolean search string, this string can be adapted, e.g., to include currently not covered interventions. However, it is also important to consider the right *comparison* arm, which is often equivalent to recommended clinical practice, as shown by the strong performance of the “known intervention” filter in the evaluation for the oesophageal cancer.

Our database of semantically interoperable guidelines, adhering to international terminology standards and classification systems, can support a variety of use cases: besides retrieval of evidence for potentially new recommendations, it can be used to retrieve evidence for existing, but consensus-based recommendations, thereby improving a guideline’s trustworthiness. Semantically interoperable metadata extracted from guidelines can also become the basis of computer-interpretable guidelines^34^, e.g., by semi-automatically populating standardized guideline representations, such as CPG-on-FHIR^35^ or other formalisms, like data-driven decision trees^36,37^. Such representations open up further applications in the domain of clinical decision support systems. Moreover, interoperable guidelines can be integrated with structured information from cancer registries^38^ or other real-world data; such integrations could not only improve guideline adoption in clinical practice, but also inform the development of future guidelines.

The dataset used for retrieval evaluation represents a rather broad notion of relevance: not all publications that are reviewed need to be considered as update signals, and not all users of the NGE system will be interested in update signals alone. However, the results suggest that different combinations of filters provided by the system can increase precision for different user groups and their specific use-cases. Due to the nature of our screening datasets, we performed a quantitative evaluation on the level of individual articles. A more relaxed evaluation scenario might incorporate articles that can be indirectly retrieved via references to registered clinical trials, which our system also incorporates. The choice of guidelines used during the evaluation of retrieval performance for update signals was determined by limited data availability: only a few guidelines from our partners were subject to an ongoing update when the project was conducted, and data on screened references were only available for a subset of those. To assess the generalizability of our results, the targeted literature search should be evaluated prospectively in future guideline updates.

Similarly, our assessment of time lags in was based on a comparatively small sample of guideline updates (22 new interventions), as only a relatively brief time span for potential updates could be considered. As of now, no historical guideline versions are available through the GGPO CMS, which constrained the availability of data to those assembled after the first GGPOnc release in 2022^25^. This small set of guidelines might not be fully representative of all update protocols encountered in practice. Although we found an overall time span of eight to ten years, which is consistent with suggestions from prior work, adding more data might provide a more reliable estimate for individual time lags.

Our current web frontend serves as a research prototype for two narrowly defined use cases, requiring further improvements and evaluations of the user experience for clinical use, and a thorough investigation of its usability using well-defined frameworks, such as the System Usability Scale^39^. Beyond evidence synthesis, there are several procedural and organizational challenges in clinical guideline development^13,40^. Delays arise from the iterative steps required to identify and prioritize key clinical (PICO) questions, conducting literature reviews, assessing the robustness of the underlying evidence, and arriving at recommendations in more or less structured ways, e.g., through the GRADE EtD framework^11^. Further complexities arise from logistical aspects, e.g., team coordination, funding constraints, managing conflicts of interest, approval processes by guideline organizations, and quality control according to international standards such as the Agree II tool^41^. A more holistic evaluation of the impact of our system should account for these factors and prospectively measure the actual improvement in guideline responsiveness to new evidence.

The NGE system relies on a few key metadata items for the retrieval of clinical trials, which imposes certain limitations. For instance, the recall with respect to the oesophageal cancer guideline update was lower than 100%, as key population items were neither explicitly mentioned in the abstract of the publication nor in its assigned MeSH terms. This information might be recovered from the full-text of the publication, which also reflects how human experts screen literature for relevance. However, it needs to be carefully evaluated whether our employed NLP components generalize well from relatively homogeneous abstracts to more diverse full-text article formats^42^. In prospective use, PubMed results are retrievable only after metadata for publication types is assigned. Although this process was recently automated by the National Library of Medicine (NLM) through the introduction of deep-learning based automated indexing using the MTIX system, a subset of publications is still subject to (slower) manual curation and quality-control^43,44^. In practice, guideline expert panels may also rely on criteria for assessing relevancy that are different from the ones currently incorporated in the system. These could include bibliographic metrics and heuristics, like journal impact factors and authorship (e.g., well-known first or last authors).

Currently, our NGE system focuses on retrieving RCTs as the gold-standard for interventional study design because we expect that results of an individual RCT might have sufficient power to change the recommended clinical practice. In practice, guideline updates are often based on existing systematic reviews or meta-analyses of multiple RCTs, which provide an even higher level of evidence. These are currently not retrieved by the system, as key NLP components, such as the PICO tagger, were trained and evaluated on RCT abstracts only. Moreover, our interpretation of significant results might have to be broadened to incorporate non-inferiority trials. In contrast, lower-level evidence (observational studies, case reports) might be more relevant for fields where large RCTs are not the norm and may not even be practical to conduct. Other types of “gray” literature, such as meeting abstracts or government documents, could be included to obtain insights in a more timely manner^45^. This might account for the present risk of publication bias when considering published results only.

The system can be extended to import additional sources of primary and synthesized evidence to the database. A possible alternative to the free resources by the NLM are, for instance, the commercial database Embase, which provides access to the latest conference abstracts and richer search functionalities compared to PubMed^46^. Furthermore, the Cochrane Central Register of Controlled Trials (CENTRAL) is an alternative library of clinical trial reports, and includes data from PubMed/Medline, and Embase, but also the proprietary Cinahl database^47^. We can consider several additional registries for the primary registration of clinical trials, e.g., maintained by the European Union (Clinical Trials Information System / CTIS) or the BfArM (German Clinical Trials Register)^48^. The WHO operates the International Clinical Trials Registry Platform (ICTRP), combining data from multiple trial registries to provide a comprehensive global view of clinical trials. Alternatives to CIViC include OncoKB^49^, My Cancer Genome^50^, and Jax-Ckb^51^. However, integration of these data sources requires clearly documented access options, e.g., through APIs^52^.

Additional guidelines could be integrated by considering other medical specialties, e.g., by applying the described German-language NLP components to all other AWMF guidelines from Germany^53^, or using comparable, language-specific or multilingual models for guidelines from other countries. However, most guidelines currently do not provide fine-grained, recommendation-level metadata in a structured format, so they would need to be extracted from many heterogeneous documents, mostly in PDF format. Similarly, English-language guidelines, e.g., from the National Comprehensive Cancer Network (NCCN) or ASCO, could be easily integrated, as high-quality biomedical Named Entity Recognition (NER) and Named Entity Normalization (NEN) solutions for English texts are widely available^54^. Some guideline management tools such as the Magic app provide similar metadata, although it involves considerable manual curation efforts^55^. As more guideline organizations adopt international interoperability standards, like CPG-on-FHIR, our system can be enriched with more guideline sources^35^.

## Methods

This section describes our incorporated methodology for obtaining an integrated database of clinical evidence, a software application for interacting with these data, and the experimental setup for the evaluation of our system.

### Data integration

**Fig. 4:**
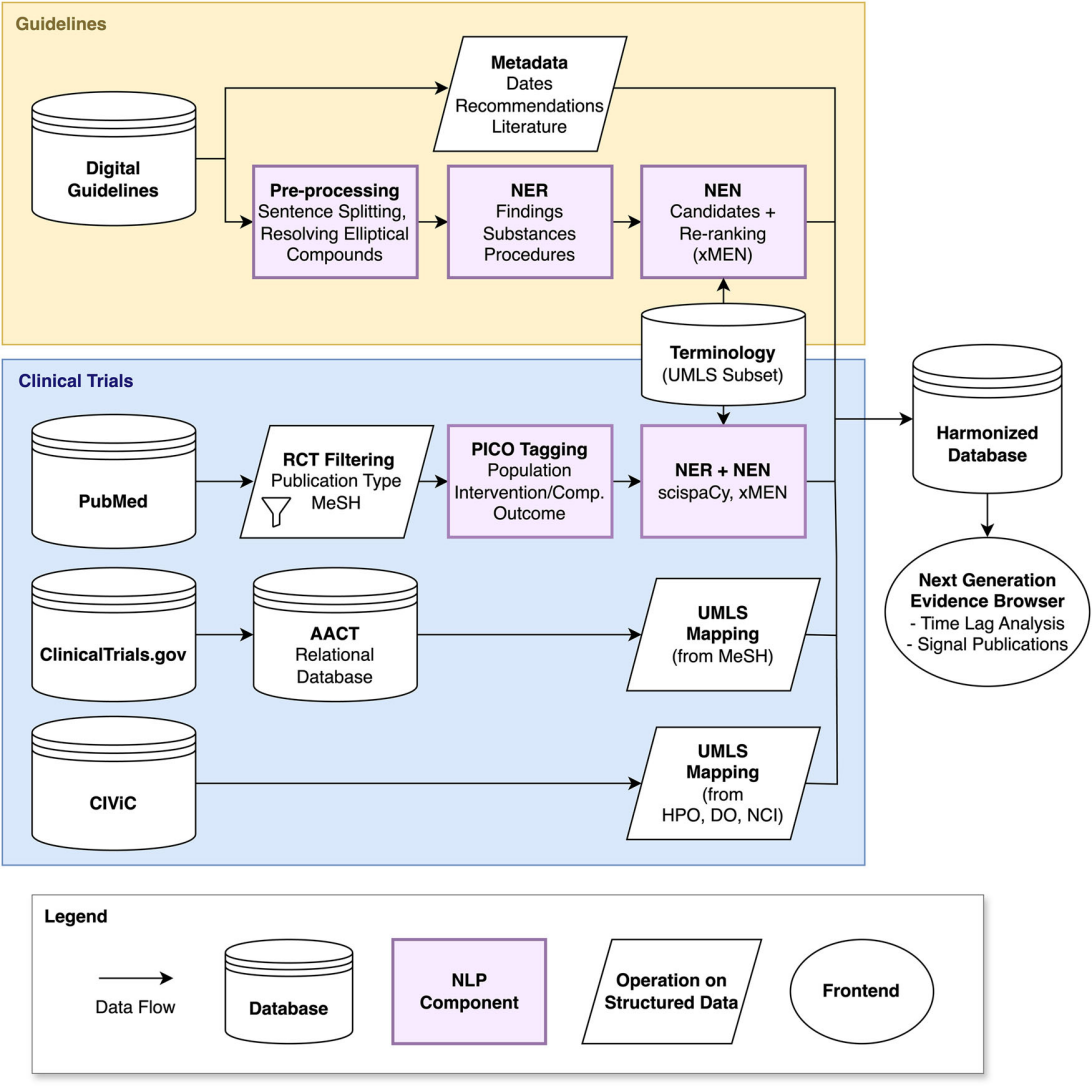
Integration of different sources of medical evidence into the harmonized NGE database. We incorporate the contents of clinical guidelines, clinical trial reports in PubMed, registered clinical trials through the Aggregate Content of ClinicalTrials.gov (AACT) database, as well as assertions from CIViC, a widely used knowledge base (KB) for precision oncology. As both guidelines and trial reports consist of mostly unstructured text content, we apply recently developed NLP components to extract structured data from these sources: this involves Named Entity Recognition (NER) and Normalization (NEN), and extraction of Population--Intervention--Comparison--Outcome (PICO) spans. All items are mapped to concept identifiers from the Unified Medical Language System (UMLS), to make them interoperable with information in structured data sources, which are coded using concepts from MeSH (Medical Subject Headings), HPO (Human Phenotype Ontology), DO (Disease Ontology), and the NCI (National Cancer Institute) thesaurus. Details on each component are provided in Table 5.

The NGE database builds upon periodically replicated copies of the underlying, heterogeneous data sources, i.e., clinical guidelines and various sources of primary evidence, such as the description and results of clinical trials. The data from each source is processed by individual Extract–Transform–Load (ETL) processes^56^. The ETL results are stored as a materialized version in a relational database. Figure 4 and Table 5 provide an overview of the involved ETL components for each of our currently integrated data sources; technical details are provided in Supplementary Table 2. Many data sources provide mostly unstructured data (clinical guidelines, PubMed abstracts). Therefore, we employ a variety of recently developed Transformer-based NLP components for NER and NEN. Since international guidelines are published in many national languages, these data require language-specific NLP tools. Presently, we focus on German oncology guidelines that we obtain from a Content Management System (CMS) maintained by the GGPO^57^. Nonetheless, our system can be extended to incorporate also other sources, e.g., the Magic app^55^ or adapted to incorporate international standards, e.g., CPG-on-FHIR^35^. For the unstructured portions of the guidelines (recommendations and background texts), we leverage NER models developed in the context of the GGPOnc project, as well as the xMEN toolkit for cross-lingual entity normalization to map these entities to CUIs from a custom, task-specific UMLS subset^25,27^. For RCT publications, we rely on curated metadata, like MeSH terms or publication types, as well as NLP-derived information from Medline titles and abstracts. To this end, we use task-specific PICO extraction models built upon the EBM-NLP corpus of Medline abstracts^58^, similar to the Trialstreamer system^59^. All NLP components are based upon fine-tuned state-of-the-art domain- and language specific encoder or encoder–decoder models, as described in Supplementary Table 2.

**Table 5:** Data sources and software components for their integration

Source	Comp.	Description	NLP Model
Clinical Guidelines	Metadata	Extraction of structured metadata from a digital guideline repository	–
	Pre-processing	Syntactic pre-processing (e.g., sentence-splitting); replacing elliptical coordinated compound noun phrases ("chemo- and radiotherapy”) with their expanded form ("chemotherapy and radiotherapy”)	Encoder–decoder model (mT5) trained with >3K manually annotated sentences in GGPOnc 2.0^26,62^
	NER	Named entity recognition for findings, substances, and procedures within clinical guidelines	Nested NER model initialized from medBERT.de and trained with >200K long, fine-grained entity annotations in GGPOnc 2.0^25,63^
	NEN	Named entity normalization of mentions to UMLS concepts	xMEN candidate generation with a knowledge base (KB) initialized from a UMLS subset, followed by re-ranking^27^
PubMed	RCT Filtering	Identification of RCTs in Medline based on publication types and MeSH terms	–
	PICO Tagging	Identification of all PICO spans within Medline abstracts	BioELECTRA model fine-tuned for PICO extraction on the EBM-NLP dataset^58,64^
	NER + NEN	Identification of all named entities within PICO spans and normalization to UMLS concepts	scispaCy NER model trained on MedMentions, scispaCy entity linker adapted to a custom UMLS subset^65,66^
Clinical-Trials.gov (via AACT)	UMLS Mapping	Mapping of already normalized conditions and interventions (MeSH terms) to UMLS concepts	–
CIViC	UMLS Mapping	Mapping of already normalized diseases (DO), phenotypes (HPO), and therapies (NCI thesaurus) to UMLS concepts	–

For each component, we indicate whether the component uses NLP and, if so, which kind of model. Please refer to Fig. 4 for a description of used abbreviations.

Mapping to UMLS CUIs ensures a high degree of semantic interoperability across overlapping items from integrated data sources. Moreover, evidence in all sources can be linked to one or more *population* and *intervention* attributes, although with different naming conventions. This mapping to populations and interventions is usually straightforward. However, the following design decisions were taken. First, *clinical drugs* and *therapeutic procedures* are considered as interventions in guidelines, based on the fine-grained named entity classes identified by the GGPOnc NER tagger^25^. Second, all *phenotypes* and *diseases* are considered as populations for CIViC.

### The NGE browser

The key use case of the NGE database is to query its content based on a clinical question, formulated with respect to population or intervention concepts, and to filter the results according to various criteria, such as publication timestamps. The database is accessible through both a REST API and the NGE browser, a user-friendly web application, which allows researchers, guideline developers, clinical practitioners, and other potential users of the system to easily interact with the data.

Figure 1 depicts the timeline view in our NGE browser, which provides a temporal perspective of the current state of clinical research on a particular intervention in the context of current and previous guideline versions. The example shows the timeline view for the drug *Ipilimumab* for the clinical indication oesophageal cancer. Results of two phase III RCTs have been published just before the literature search for the latest update (version 4.0) has been performed. As a result, these were subject to the screening and data extraction phase of the review process and included in the guideline (green stars in the figure). However, shortly after finishing the search, two additional reports of phase III RCTs were published. Moreover, new data (subgroup analyses) for the two RCTs that were included were published in the meantime.

Figure 2 depicts the search view of our NGE browser, which allows retrieving RCT publications and trial register entries based on a particular population, and filtered according to different selection criteria such as publication dates or clinical trial phases. The user has to select at least a population, here given by a guideline topic. When using the system without the graphical user interface but through its exposed REST API, it can also be queried with custom sets of UMLS concepts, e.g., to find evidence for a specific subpopulation. The result is a list of RCTs from different sources, i.e., PubMed articles retrieved through Medline or CIViC, or clinical trials registered at ClinicalTrials.gov. To guide the user to the relevant search results, the tool highlights extracted interventions using color codes based on their occurrence within the selected guideline that is related to the selected publication. The search view offers various selection criteria to filter the result set according to different requirements a user may have. The default values are tailored to a prospective scenario, i.e., targeted for users, who wish to identify new, potentially practice-changing evidence with respect to an existing clinical guideline. A detailed description of the available filters is provided in Supplementary Fig. 6 and Supplementary Table 3. Note that all filter criteria are pre-computed as structured metadata upon import into the database, so there is no computational overhead of combining filters, but also no interaction between NLP models. In the following, we focus on two main innovative features enabled by our employed NLP components.

The first novel selection criteria stem from contextualizing the search with the current state of recommended clinical practice represented by the guidelines in the database. As highlighted in Fig. 2, interventions mentioned in a clinical trial can have different relationships to a guideline: they might be mentioned (a) anywhere in the guideline, (b) inside a recommendation, or (c) not mentioned at all. We hypothesize that interventions that are not yet recommended or mentioned otherwise provide particularly strong update signals for an existing guideline.

We further suspect that the results from RCTs might be of particular importance when they report a significant improvement of some outcome of interest, especially for new interventions. Therefore, a flag is included to filter trials based on the statistical significance of their findings. In ClinicalTrials.gov, this information is often available as part of the structured results: here, we consider any RCT with a change in outcome associated with a *p* value lower than 0.05 as significant. For RCT reports in PubMed, the required details are obtained from the free-text abstract using a binary text classifier. Our employed classifier was obtained by fine-tuning a PubMedBERT model with annotations derived from the Evidence Inference 2.0 dataset^60,61^. This feature is disabled by default because it is considered as highly experimental. More details can be found provided in Supplementary Table 3.

### Evaluation dataset for time lag analysis

Our evaluation dataset for analyzing time lags is based upon GGPO guidelines that have received an update in the timeframe 2022–2024, i.e., between the GGPOnc releases 2.0 and 2.3^25^. Using the UMLS-normalized entity mentions and recommendation metadata, we identify new interventions, which have been recommended for the first time in any of the updated guidelines within the given time frame. In addition, the following filtering steps are applied, to ensure that the data is of sufficiently high quality for further analysis:

1. NEN confidence after re-ranking of at least 0.1,
2. Exclusion of generic interventions such as “chemotherapy”,
3. CUI belongs to the UMLS semantic network hierarchy “Pharmacologic Substance” (TUI: T121) or “Therapeutic or Preventive Procedure” (T061), and
4. At least one clinical trial with this intervention can be found in our NGE system.

Step 1 excluded many non-pharmacological interventions, as these are more challenging to normalize with high confidence. Step 4 excludes some genuine interventions such as “meditation-based stress reduction” or “Yoga” for endometrial cancer, where the current guideline recommendation is based on the cross-sectional guideline on complementary medicine, rather than individual trials for the particular combination of intervention and population.

### Evaluation datasets for signal retrieval

The characteristics of two evaluation datasets for retrieval performance are presented in Table 2. For the oesophageal cancer guideline update, the complete full-text and abstract screening decisions were provided. Out of 3147 total references in the time frame from January 2019 to April 2022, 139 were duplicates retrieved from different sources (e.g., PubMed or the Cochrane database). Another 2741 references were excluded during title–abstract screening, and 195 additional ones after full-text screening. However, nine excluded references were already cited in a previous guideline version, which is possible due to overlaps of the search time frame with previous minor updates (version 3.1). If these references were retrieved by the NGE system, they would (arguably) be regarded as relevant; therefore, we consider them in the evaluation. A total of 81 references were included, but since the system is designed to retrieve only RCTs, only the subset of 26 RCTs is considered for evaluation (line “RCTs included” in Table 2). Out of the 2927 excluded references, 290 were RCTs ("RCTs excluded”). The search retrieved additional results that were included in the datasets, as described in the Results section. 15 additional references were considered for oesophageal cancer following another manual review (9 included, 6 excluded).

For Hodgkin lymphoma, the screening period overlapped with the screening period of the former guideline update. Hence, the NGE system retrieves additional results that were already incorporated in the previous guideline version; they were manually marked as included. These results constitute the final ground-truth for system evaluation (lines “RCTs included / excluded (final)”). For the Hodgkin lymphoma dataset, references excluded during title–abstract screening are not available. Therefore, this dataset contains only 168 references, which were subject to full-text screening. Out of these, 105 were excluded, and two were added as being already cited in the current Hodgkin lymphoma guideline. From the remaining 65 references, 25 were RCTs, which were complemented by manually reviewed references. Since the results from title–abstract exclusion are missing, only 35 excluded RCTs are available for the evaluation of the system regarding the Hodgkin lymphoma update.

## Supplementary information

Supplementary Information

## Data Availability

Recent versions of German oncology guidelines from the GGPO are available as GGPOnc releases on Zenodo: https://zenodo.org/records/12520623/. Baseline and daily update dumps from PubMed can be downloaded directly from the NLM: https://pubmed.ncbi.nlm.nih.gov/download/. Monthly dumps from ClinicalTrials.gov can be downloaded through the AACTproject: https://aact.ctti-clinicaltrials.org/download/. Nightly dumps from CIViC can be downloaded from the CIViC website: https://civicdb.org/releases/main/. The literature screening datasets for the oesophageal cancer and Hodgkin lymphoma guideline updates can be made available upon request.

## Code availability

The source code to create a local instance of the NGE system is available as open-source software on GitHub: https://github.com/hpi-dhc/nge_db/. Experimental results can be reproduced using the notebooks and software versions indicated in the repository. A prototype of our NGE web application is online available at: https://we.analyzegenomes.com/nge/.
