# Supplementary material for: High-precision information retrieval for rapid clinical guideline updates
Source: NPJ Digit Med. 2025 Apr 27;8:227. doi: 10.1038/s41746-025-01648-5 (PMC12034796; doi:10.1038/s41746-025-01648-5)
Supplement: Supplementary file 1 — Supplementary Information [file 41746_2025_1648_MOESM1_ESM.pdf]

Supplementary Information

Guideline	Update	New Interventions
Endometrial cancer	1.0 ▶ 2.0	Trastuzumab, Dostarlimab, Pembrolizumab
Hepatocellular and biliary cancer	3.0 ▶ 4.0	Durvalumab, Tremelimumab
Lung cancer	1.0 ▶ 2.0	Durvalumab, Cemiplimab, Nivolumab + Ipilimumab, Amivantamab, Lorlatinib, Entrectinib, Repotrectinib, Larotrectinib, Selpercatinib, Pralsetinib, Capmatinib, Sotorasib
Oesophageal cancer	3.0 ▶ 3.1	Nivolumab, Pembrolizumab
Pancreatic cancer	2.0 ▶ 3.0	Low-molecular-weight heparin
Prostate cancer	6.2 ▶ 7.0	Niraparib, Talazoparib

Supplementary Table 1 Newly recommended interventions per oncology guideline within the considered timeframe of 2022–2024 (n=22)

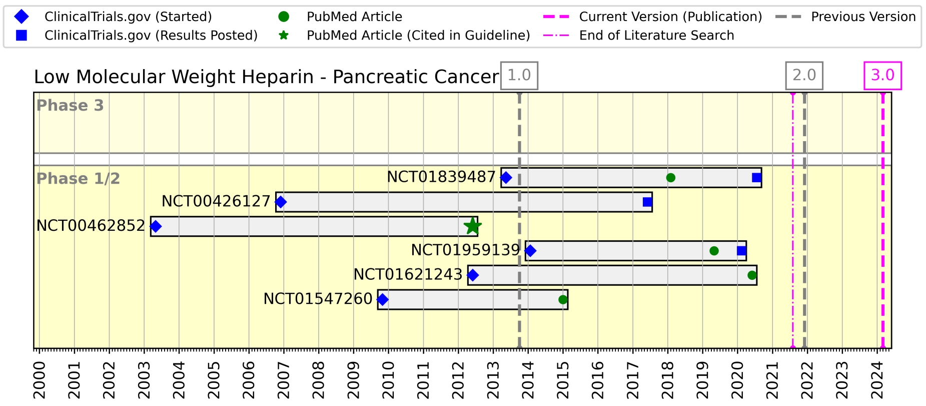

Supplementary Figure 1 Timeline of clinical trials concerning the intervention "Low Molecular Weight Heparin" (LMWH, UMLS CUI: C0019139) for pancreatic cancer. It took almost nine years for the first trial on *Dalteparin*, a specific LMWH, for pancreatic cancer patients to be completed [1]. Additionally, it took another 12 years until anticoagulation was introduced as a topic in version 3.0 of the guideline.

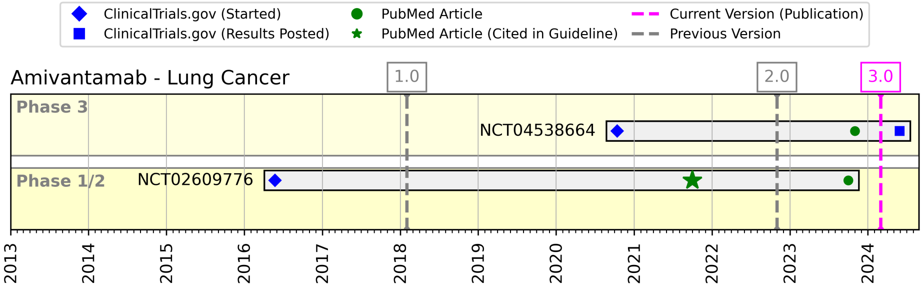

Supplementary Figure 2 Timeline of clinical trials investigating "Amivantamab" (C5446297) for lung cancer. Amivantamab was first recommended in version 2.0 of the guideline, based on early results of a phase I trial [2], while a phase III trial was still ongoing [3]. Note that there was no specified date for literature search completion in version 3.0.

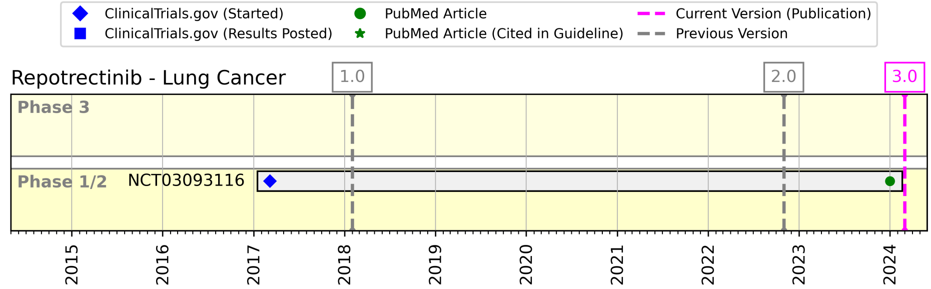

Supplementary Figure 3 Timeline of clinical trials investigating "Repotrectinib" (C4524909) for lung cancer. Repotrectinib was recommended based on an ongoing phase I/II trial; in this case, the guideline refers to a conference abstract (not included in the NGE database), as no peer-reviewed publication was available until very recently [4, 5].

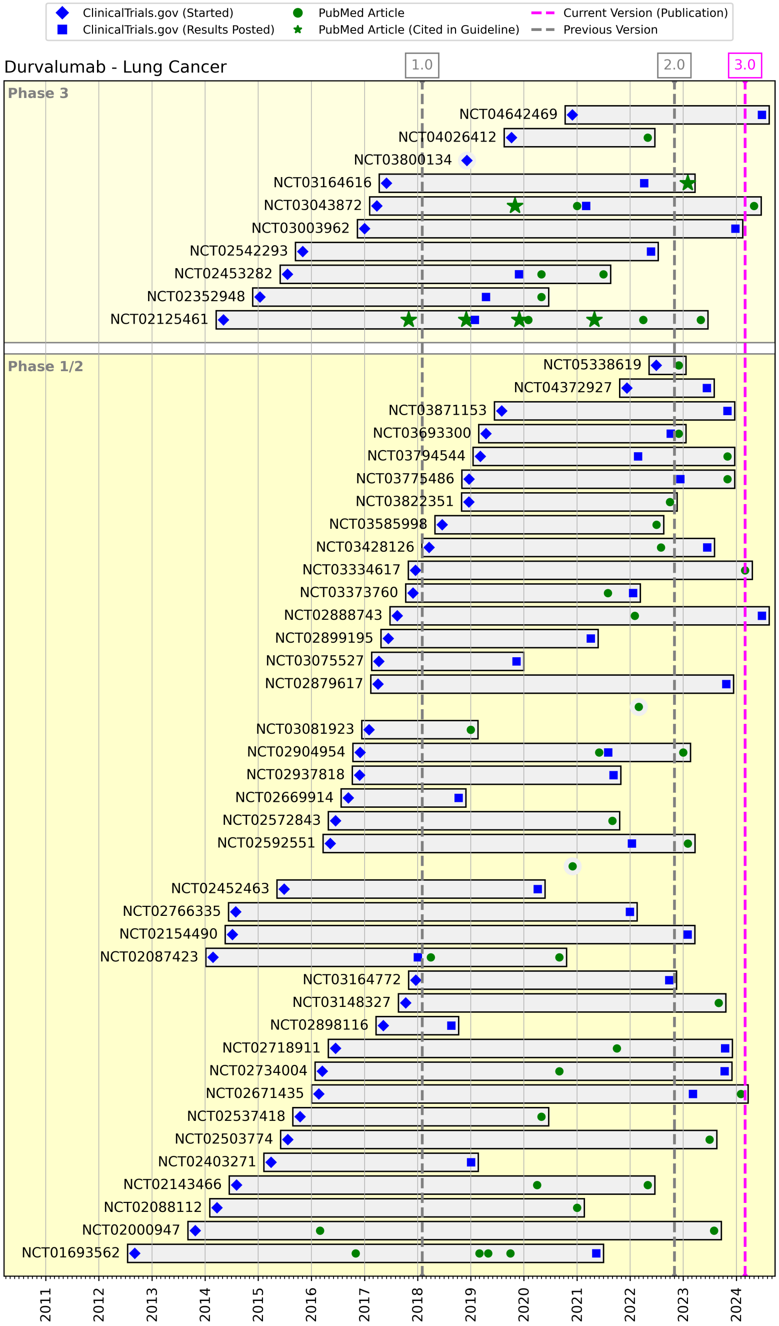

Supplementary Figure 4 Timeline of clinical trials investigating "Durvalumab" (C4055109) for lung cancer. The recommendation was included in version 2.0, almost 5 years after results of the first phase III trial have been published. This can be explained by the long update interval between lung cancer guideline version 1.0 and 2.0, which spanned around 4.5 years. The first *Durvalumab* phase III trial results [6] were published just before the final publication of the 1.0 release, and were thus not included in the systematic literature search for this version.

Source	Description
Clinical Guidelines (GGPO CMS)	Recent versions of clinical guidelines extracted from the Content Management System (CMS) of the GGPO [7], as described by Borchert et al. [8]. The structured metadata (e.g., timestamps, versions, recommendation metadata, and literature references) need no further processing, and can be stored directly in the database according to their respective data types. In contrast, the textual guideline contents (recommendations and background texts) are used as inputs to an NLP pipeline for German medical documents. 1) compound noun phrases are resolved, such that entities within elliptical compounds can be more easily detected and normalized [9]. 2) NER tagging is applied using a model for long, fine-grained, nested entity spans, initialized from MEDBERT.DE [10]. 3) The identified mentions are normalized to UMLS CUIs with xMEN [11]. The target KB for NEN is initialized from a subset of the UMLS containing the source vocabularies SNOMED CT, MESH, MEDDRA, and the NCI, and its German versions, if available. 4) a weakly supervised re-ranker is employed, i.e., a cross-encoder model trained on a machine-translated version of MEDMENTIONS [12]. The populations for each guideline are not explicitly defined with respect to any terminology system; a formal definition of a guideline's key questions according to PICO criteria is planned for future versions of the GGPO CMS. We manually identify one or multiple CUIs as the root population of a guideline. For instance, the scope of the guideline for "prostate cancer" can be defined by the single concept "Prostatic Neoplasms" (CUI: C0033578), while the guideline for "Oro- and hypopharyngeal carcinoma" needs to be defined in terms of two concepts "Oropharyngeal Neoplasms" (C0029295) and "Hypopharyngeal Neoplasms" (C0020627). Upon import into the database, these root population CUIs are resolved to descendant concepts within the UMLS (exploiting the *narrower* (RN) / *child* (CD) relationships in the UMLS artifact `MRREL`), as shown in Supplementary Figure 5.
PubMed (RCT Reports)	MEDLINE abstracts of RCT reports and their metadata, which are available as daily updated dumps from a file server maintained by the NLM. To detect RCTs among the downloaded MEDLINE articles, the NGE system relies on MEDLINE metadata for publication types and MESH terms. The included publication types are: "Randomized Controlled Trial", "Clinical Trial, Phase I", "Clinical Trial, Phase II", and "Clinical Trial, Phase III". Abstracts with the MESH term "Randomized Controlled Trials as Topic" are also included.<br>In the downloaded RCT abstracts, PICO spans are identified using a BIOELECTRA model, which was fine-tuned on the PICO span extraction task in the EBM-NLP corpus [13]. The pre-trained model from Kanakarajan et al. [14] is available on the HUGGING FACE Hub [15]. Within these PICO spans, all medical named entities are identified using the SCISPACY NER pipeline (`en_core_sci_lg`). In addition, we rely on the integrated SCISPACY linker for NEN, which is equivalent to the TF-IDF linker in xMEN. However, the default SCISPACY linker is re-configured to use the same UMLS subset introduced for normalizing mentions in GGPONC. This way, all named entities are normalized to UMLS CUIs, and it can be inferred whether they refer to an intervention, population, or outcome, according to their overlap with PICO spans.
ClinicalTrials.gov (Registered Trials)	Information about a large number of (finished and ongoing) clinical trials provided by the NLM. The data includes comprehensive information on eligibility and exclusion criteria, interventions, and study arms. For many trials, their results are also published in a structured format. Instead of accessing the data directly from CLINICALTRIALS.GOV, we rely on a tabular representation of the data provided through a monthly dump of the AACT database [16]. Interventions and conditions in AACT are already indexed with MESH terms, which can be easily mapped to UMLS CUIs through the UMLS metathesaurus. Moreover, the NLM maintains references between publications in MEDLINE and registered trials, which are incorporated into the database.
CIViC (Precision Oncology Knowledge Base)	Assertions from the Clinical Interpretation of Variants in Cancer (CIVIC) database, a crowdsourced community resource for the clinical actionability of cancer variants [17]. A recent survey [18] revealed that CIVIC is among the most widely used KB in clinical practice with convenient access options, e.g., through nightly dumps of the full database content and an API. For data extraction, we use the PYTHON library CIVICPY for downloading contents of the database [19]. Conceptually, the results are treated equivalently to clinical trials from PUBMED; however, for results in CIVIC, the system relies on curated rather than NLP-derived metadata.

Supplementary Table 2 Implementation details on the integrated data sources and employed ETL components, in particular those based on NLP models.

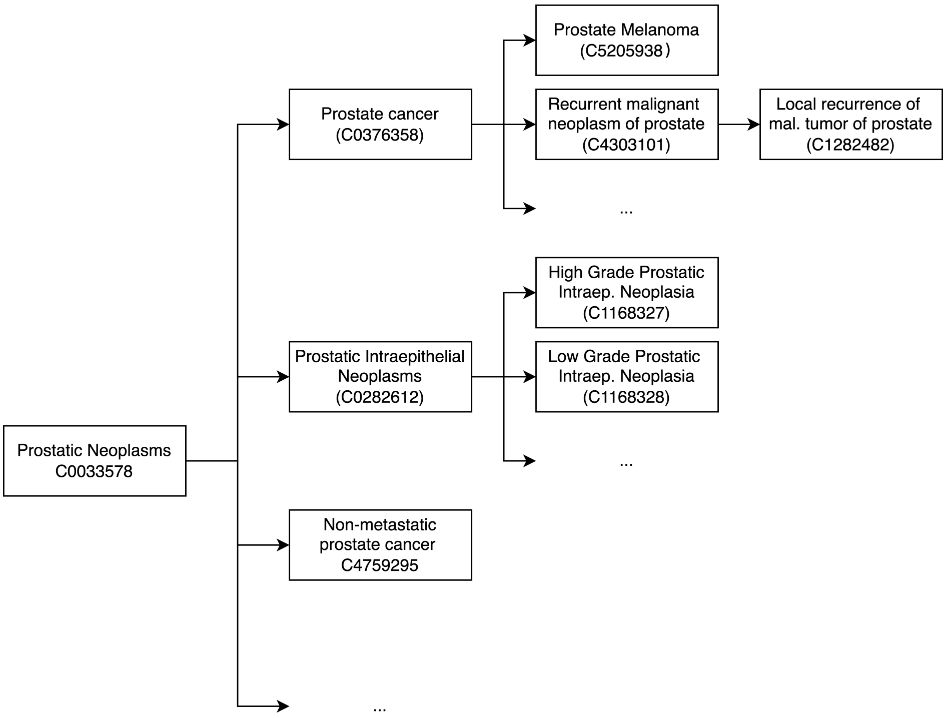

Supplementary Figure 5 Resolving top-level population Concept Unique Identifiers (CUIs) for a guideline topic to its descendants. To this end, we use child-parent-relationships of the hierarchy modelled by the UMLS.

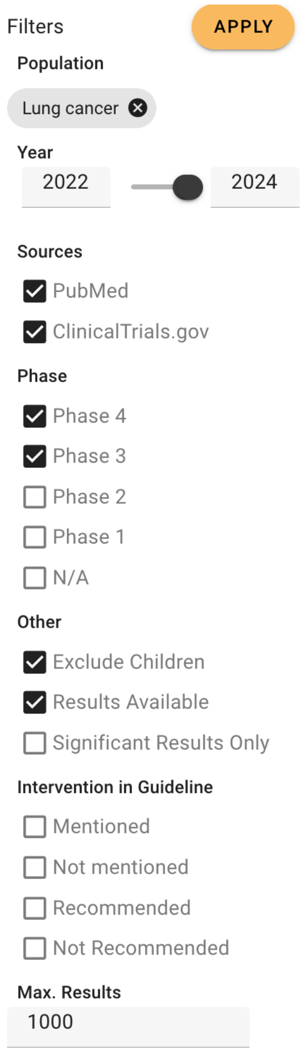

Supplementary Figure 6 Selection criteria for trial search in the NGE database. Screenshot from the web interface of the NGE Browser.

**Criterion**	**Description**
Population	The minimum input a user needs to provide. Technically, this is a set of CUIs, from which at least one has to be present in the populations assigned to a clinical trial to be included in the result set. In the current user interface, the user can select a guideline topic as a proxy for a set of CUIs. For each topic, this set is pre-computed using the hierarchical expansion shown in Supplementary Figure 5.
Publication Date	For most use cases, the publication date of a trial is relevant, as recent results are usually more interesting than old ones. Moreover, for most guideline updates, there is a fixed date when the literature search has been completed. This date is essential for the evaluation of the system later in this chapter: in a retrospective setting, the result set should be limited to not include results past this date. In a prospective scenario, it is more desirable to find all evidence after this cutoff, which is potentially relevant for the guideline update, but could not be not covered by a previous literature screening. The default range for this filter is set to two years prior to the publication date of the selected guideline topic (minimum), and the current date (maximum).
Sources	Users can select the sources to be included in the result set, e.g., only include data from PubMed or ClinicalTrials.gov. Currently, only published, peer-reviewed reports of clinical trials are considered during guideline updates. However, for different surveillance strategies, ongoing clinical trials or results posted on ClinicalTrials.gov prior to a journal publication might be equally relevant.
Phase	Results can be filtered by the extracted trial phase; by default, only phase III and IV trials are returned, as these are the most relevant for the prospective scenario. Unfortunately, the phase is not unambiguously defined as metadata in the integrated sources. Therefore, a heuristic is applied to extract the trial phase, using a regular expression to extract numerical values in the following order: publication titles (PubMed), publication types, MeSH terms, and abstract / description. This heuristic has high recall, but might lead to some false positives, e.g., when an abstract mentions a prior trial of a lower phase. Many trials in oncology are combined phase I/II trials. For these reasons, a trial might have multiple phases assigned in the result set.
Interventions	Similar to the expansion of population CUIs shown in Supplementary Figure 5, matching of interventions in trials to guidelines also accounts for potential child–parent–relationships. Thus, an intervention is also considered present in a guideline, if any of its children are already mentioned. For instance, when a guideline mentions "Cetuximab" (C0995188), its parents "Monoclonal Antibodies" (C0003250) or "Protein Kinase Inhibitors" (C1449702) are also considered as already known.
Significant Results	For results from ClinicalTrials.gov, this information can be inferred from the structured results tab: any trial with a change in outcome associated with a p value lower than 0.05 is considered significant. For published trial reports, this information needs to be obtained from the free-text abstract. To this end, we use a binary text classifier, which was trained by fine-tuning PubMedBERT [20] on a dataset derived from annotations in the Evidence Inference 2.0 dataset [21]. The classifier achieves an F_1 score of 0.84 for classifying trials reporting significant effects on the Evidence Inference test set (precision: 0.86, recall: 0.80).
Other Criteria	As most guidelines explicitly exclude childhood cancers from their scope, the search excludes trials related to children by default, i.e., by filtering by occurrence of the concept "Child" (C0008059) within populations (usually assigned as a MeSH term in PubMed). Moreover, only results from ClinicalTrials.gov with published results are included by default; when this filter is disabled, ongoing trials or completed trials without posted results are included as well, which tends to increase the result set substantially.

Supplementary Table 3 Detailed description of the search criteria available in the NGE browser.

Supplementary References

[1] Maraveyas, A., Waters, J., Roy, R., Fyfe, D., Propper, D., Lofts, F., *et al.*: Gemcitabine versus gemcitabine plus dalteparin thromboprophylaxis in pancreatic cancer. European journal of cancer **48**(9), 1283–1292 (2012)

[2] Park, K., Haura, E.B., Leighl, N.B., Mitchell, P., Shu, C.A., Girard, N., *et al.*: Amivantamab in egfr exon 20 insertion–mutated non–small-cell lung cancer progressing on platinum chemotherapy: initial results from the chrysalis phase i study. Journal of Clinical Oncology **39**(30), 3391–3402 (2021)

[3] Zhou, C., Tang, K.-J., Cho, B.C., Liu, B., Paz-Ares, L., Cheng, S., *et al.*: Amivantamab plus chemotherapy in nsclc with egfr exon 20 insertions. New England Journal of Medicine **389**(22), 2039–2051 (2023)

[4] Cho, B.C., Drilon, A.E., Doebele, R.C., Kim, D.-W., Lin, J.J., Lee, J., *et al.*: Safety and preliminary clinical activity of repotrectinib in patients with advanced ros1 fusion-positive non-small cell lung cancer (trident-1 study). J Clin Oncol **37**(15_suppl), 9011 (2019)

[5] Drilon, A., Camidge, D.R., Lin, J.J., Kim, S.-W., Solomon, B.J., Dziadziuszko, R., *et al.*: Repotrectinib in ros1 fusion–positive non–small-cell lung cancer. New England Journal of Medicine **390**(2), 118–131 (2024)

[6] Antonia, S.J., Villegas, A., Daniel, D., Vicente, D., Murakami, S., Hui, R., *et al.*: Durvalumab after chemoradiotherapy in stage iii non–small-cell lung cancer. New England Journal of Medicine **377**(20), 1919–1929 (2017)

[7] Seufferlein, T., Kopp, I., Post, S., Jonat, W., Kreienberg, R., Nothacker, M., *et al.*: Onkologische Leitlinien: Herausforderungen und zukünftige Entwicklungen. Forum **34**, 277–283 (2019)

[8] Borchert, F., Lohr, C., Modersohn, L., Witt, J., Langer, T., Follmann, M., Gietzelt, M., Arnrich, B., Hahn, U., Schapranow, M.-P.: GGPONC 2.0 - the German clinical guideline corpus for oncology: Curation workflow, annotation policy, baseline NER taggers. In: Proceedings of the Language Resources and Evaluation Conference (LREC), pp. 3650–3660. European Language Resources Association, Marseille, France (2022)

[9] Kämmer, N., Borchert, F., Winkler, S., Melo, G., Schapranow, M.-P.: Resolving elliptical compounds in German medical text. In: The 22nd Workshop on Biomedical Natural Language Processing and BioNLP Shared Tasks, pp. 292–305. Association for Computational Linguistics, Toronto, Canada (2023)

[10] Bressem, K.K., Papaioannou, J.-M., Grundmann, P., Borchert, F., Adams, L.C., Liu, L., Busch, F., Xu, L., Loyen, J.P., Niehues, S.M., Augustin, M., Grosser, L., Makowski, M.R., Aerts, H.J.W.L., Löser, A.: medBERT.de: A comprehensive German BERT model for the medical domain. Expert Systems with Applications **237**, 121598 (2024)

[11] Borchert, F., Llorca, I., Roller, R., Arnrich, B., Schapranow, M.-P.: xMEN: A

modular toolkit for cross-lingual medical entity normalization. arXiv [cs.CL] (Currently under review, pre-print available) **2310.11275** (2023)

[12] Mohan, S., Li, D.: MedMentions: A large biomedical corpus annotated with UMLS concepts. In: Automated Knowledge Base Construction (AKBC) (2019)

[13] Nye, B., Li, J.J., Patel, R., Yang, Y., Marshall, I., Nenkova, A., *et al.*: A corpus with multi-level annotations of patients, interventions and outcomes to support language processing for medical literature. In: Proceedings of the 56th Annual Meeting of the Association for Computational Linguistics (Volume 1: Long Papers), pp. 197–207. Association for Computational Linguistics, Melbourne, Australia (2018)

[14] Kanakarajan, K.r., Kundumani, B., Sankarasubbu, M.: BioELECTRA:pretrained biomedical text encoder using discriminators. In: Proceedings of the 20th Workshop on Biomedical Language Processing, pp. 143–154. Association for Computational Linguistics, Online (2021). Foo

[15] Kanakarajan, K.R.: kamalkraj/BioELECTRA-PICO. https://huggingface.co/kamalkraj/BioELECTRA-PICO [retrieved: Nov 1, 2024] (2021)

[16] (CTTI), C.T.T.I.: Aggregate Analysis of ClinicalTrials.gov (AACT) Database. https://aact.ctti-clinicaltrials.org/ [retrieved: Nov 1, 2024] (2024)

[17] Griffith, M., Spies, N.C., Krysiak, K., McMichael, J.F., Coffman, A.C., Danos, A.M., *et al.*: Civic is a community knowledgebase for expert crowdsourcing the clinical interpretation of variants in cancer. Nature genetics **49**(2), 170 (2017)

[18] Borchert, F., Mock, A., Tomczak, A., Hügel, J., Alkarkoukly, S., Knurr, A., Volckmar, A.-L., Stenzinger, A., Schirmacher, P., Debus, J., Jäger, D., Longerich, T., Fröhling, S., Eils, R., Bougatf, N., Sax, U., Schapranow, M.-P.: Knowledge Bases and Software Support for Variant Interpretation in Precision Oncology. Briefings in Bioinformatics **22**(6) (2021)

[19] Wagner, A.H., Kiwala, S., Coffman, A.C., McMichael, J.F., Cotto, K.C., Mooney, T.B., *et al.*: Civicpy: a python software development and analysis toolkit for the civic knowledgebase. JCO Clinical Cancer Informatics **4**, 245–253 (2020)

[20] Gu, Y., Tinn, R., Cheng, H., Lucas, M., Usuyama, N., Liu, X., *et al.*: Domain-specific language model pretraining for biomedical natural language processing. ACM Transactions on Computing for Healthcare (HEALTH) **3**(1), 1–23 (2021)

[21] DeYoung, J., Lehman, E., Nye, B., Marshall, I., Wallace, B.C.: Evidence inference 2.0: More data, better models. In: Proceedings of the 19th SIGBioMed Workshop on Biomedical Language Processing, pp. 123–132. Association for Computational Linguistics, Online (2020)
